# Allele specific chromatin signals, 3D interactions, and motif predictions for immune and B cell related diseases

**DOI:** 10.1038/s41598-019-39633-0

**Published:** 2019-02-25

**Authors:** Marco Cavalli, Nicholas Baltzer, Husen M. Umer, Jan Grau, Ioana Lemnian, Gang Pan, Ola Wallerman, Rapolas Spalinskas, Pelin Sahlén, Ivo Grosse, Jan Komorowski, Claes Wadelius

**Affiliations:** 1 Science for Life Laboratory, Department of Immunology, Genetics and Pathology, Uppsala University, Uppsala, Sweden; 2 Department of Cell and Molecular Biology, Computational Biology and Bioinformatics, Uppsala University, Uppsala, Sweden; 3 Institute of Computer Science, Martin Luther University Halle-Wittenberg, Halle, Germany; 4 German Centre for Integrative Biodiversity Research (iDiv) Halle-Jena-Leipzig, Leipzig, Germany; 5 Science for Life Laboratory, Division of Gene Technology, KTH Royal Institute of Technology, Stockholm, Sweden; 6 Institute of Computer Science, Polish Academy of Sciences, Warszawa, Poland

## Abstract

Several Genome Wide Association Studies (GWAS) have reported variants associated to immune diseases. However, the identified variants are rarely the drivers of the associations and the molecular mechanisms behind the genetic contributions remain poorly understood. ChIP-seq data for TFs and histone modifications provide snapshots of protein-DNA interactions allowing the identification of heterozygous SNPs showing significant allele specific signals (AS-SNPs). AS-SNPs can change a TF binding site resulting in altered gene regulation and are primary candidates to explain associations observed in GWAS and expression studies. We identified 17,293 unique AS-SNPs across 7 lymphoblastoid cell lines. In this set of cell lines we interrogated 85% of common genetic variants in the population for potential regulatory effect and we identified 237 AS-SNPs associated to immune GWAS traits and 714 to gene expression in B cells. To elucidate possible regulatory mechanisms we integrated long-range 3D interactions data to identify putative target genes and motif predictions to identify TFs whose binding may be affected by AS-SNPs yielding a collection of 173 AS-SNPs associated to gene expression and 60 to B cell related traits. We present a systems strategy to find functional gene regulatory variants, the TFs that bind differentially between alleles and novel strategies to detect the regulated genes.

## Introduction

More than 15% of the variants reported today in the Genome Wide Association Studies (GWAS) catalog are associated to immune system diseases. It is today established that the top hits in GWAS rarely drive the associations^1^ likely due to heterogeneity in and between the study groups e.g. in rare functional variants. In addition, since the target genes are often not obvious from the associations, most of the molecular mechanisms behind the genetic contributions to immune and autoimmune diseases still remain poorly understood.

In recent years, the study and better understanding of the complexity of autoimmune diseases has prompted a shift from an almost exclusively T cell mediated view to a more synergistic view with a prominent role for B cells. Several functions mediated by B cells, such as secretion of autoantibodies, inflammatory cytokines, presentation of autoantigens, modulation of antigen processing etc., are today consistently reported as central in the onset of several autoimmune diseases^2^.

Regulatory B cells^3^ are nowadays gaining a prominent role in explain the etiology of systemic lupus erythematosus (SLE) characterized by the production of antinuclear antibodies; rheumatoid arthritis (RA) a chronic inflammation of the joint capsule and synovial membrane; multiple sclerosis (MS) characterized by multifocal inflammation, demyelination, gliosis and axonal loss in the central nervous system (CNS); inflammatory bowel disease (IBD), a chronic relapsing intestinal inflammatory disease classified into two major forms, Crohn’s disease (CD) and ulcerative colitis (UC); type 1 diabetes (T1D), an autoimmune disease in which insulin-producing β-cells in the pancreatic islets are destroyed and many more autoimmune, allergic and socially impairing diseases (e.g. vitiligo, psoriasis, atopic dermatitis).

The majority of genes show difference in activity between people and it has been proposed that a majority of drivers of GWAS signals are located in non-coding regulatory elements and affect the binding of transcription factors (TFs) leading to allelic difference in expression^4^. Therefore, linking genomic variation to diseases or phenotype is a complex process that involves three major steps: (i) identify the causal gene regulatory variant(s), (ii) identify the TF(s) that bind to the variants, (iii) identify the target gene(s) whose deregulation lead to the phenotype. This opens the field for functional studies of the biological mechanisms of disease.

The majority of the GWAS top associated variants are located in non-coding regions and often in high linkage disequilibrium (LD) with several other variants making it difficult to pinpoint the real functional SNP(s). One way to find putative functional variants is to detect regions with allele specific (AS) binding of TFs or their surrogates histone modifications, suggesting a different regulatory downstream role based on the individual genotypes. A powerful way to do this is to study the heterozygous positions in a cell/tissue so that one allele acts as the internal control for the other. A limitation is that when studying one person you only survey some common variants and a small fraction of all rare ones. However, if you study the same cell/tissue in several individuals you cover a larger fraction of common variants, which are the relevant ones for GWAS studies. ChIP-seq data for TFs and histone modifications provide snapshots of direct and indirect protein-DNA interactions allowing the identification of heterozygous SNPs with significant allele-specific signals (AS-SNPs).

The central idea is that a regulatory functional variant can change a TF binding site resulting in altered gene regulation. After our original discovery that AS signals can be detected using ChIP^5^ and ChIP-seq^6^, this concept has been exploited by several bioinformatics approaches such as sTRAP^7^, rSNP MAPPER^8^, AlleleSeq^9^, iASeq^10^ and ALEA^11^ that have been developed to use RNA-seq and ChIP-seq datasets in order to gather information about allele specific expression (ASE) and binding (ASB). Allele specific variants have also been detected using data from DNaseI hypersentitive sites (DHSs) analyses^12^.

We have previously shown^6^ that interrogating ASB events in one cell line is limited to the 33% heterozygous common SNPs defined by the Hardy-Weinberg equilibrium. Moreover, we demonstrated how a small set of ChIP-seq experiments for TFs allows the detection of most common AS-SNPs. This approach aimed at prioritizing candidate functional variants has proven quite robust with up to 70% of the AS-SNPs tested via luciferase assays in HepG2 cells showing an allele specific enhancer activity^13^. Here we present the results of the identification of SNPs that show allele-specific behavior using a minimal set of ChIP-seq datasets produced for three histone modifications and two genome architectural proteins in a population of seven B cell lines where the likelihood of finding heterozygous variants with an allele frequency (AF) of 0.35–0.65 in at least one individual is 99%, greatly increasing the pool of heterozygous common SNPs that can be investigated for AS signals. In this set of cell lines we interrogate 85% of common genetic variants in the population for potential regulatory effect. Increasing the population size is also beneficial toward the identification of rare AS-SNPs, but the huge number of rare variants in the human population means that only a small fraction of them is heterozygous when analyzing seven individuals.

One traditional approach to identify the DNA-binding TF is to utilize databases such as TRANSFAC^14^, JASPAR^15^, UniPROBE^16^ or HOCOMOCO^17^ that provide experimentally proven binding sites (BSs) and motif models typically represented by positional weight matrices (PWMs). DNA sequences of interest are then matched against these PWMs to predict which TF may putatively bind the selected sequence. One limitation of PWM models is that they neglect intra-motif dependencies, so motif models that take into account intra-motif dependencies such as Markov Models^18,19^, Transcription Factor Flexible Models (TFFMs)^20^, Variable-Order Markov Models (VOMMs)^21^, or Parsimonious Markov Models (PMMs)^22^ have been developed for an improved prediction of BSs. These motif models take into account intra-motif dependencies by considering conditional probabilities where the probability of the nucleotide at the current motif position may depend on the previous k nucleotides. For traditional Markov Models, the number of parameters at each motif position increases exponentially with the order k, which may lead to overfitting and poor prediction. In contrast, TFFMs, VOMMs, and PMMs address this problem by a variable choice of subsets of nucleotides considered as conditioning variables in a position-specific manner and have been shown to outperform PWM models in the context of motif discovery^23,24^. However, these motif models do not lead to an improved prediction of BSs for every TF, but one noteworthy feature of these motif models is that in the presence of intra-motif dependencies the improvement is typically substantial, whereas in the absence of intra-motif dependencies the decrease is typically minor or not measurable, so that utilizing these motif models, as in the present study, can be said to be often potentially beneficial and almost never potentially harmful^23,24^.

Finally, the question remains on how to identify the target gene(s) controlled by the regulatory element. Historically the closest gene to a GWAS SNP was considered the likely candidate. While this holds true in many cases^25^, variants are also located in intergenic cis-acting enhancer regions that can act at a distance of tens or hundreds of kb, precluding an easy prediction of the target gene(s). Promoter-enhancer interactions are often restricted to specific regions by the TF CTCF, which together with SA.1 and other proteins in the cohesin complex creates topologically associated domains (TADs). Allele specific behavior of CTCF and SA.1 may change domain size and thereby different promoter-enhancer interactions may occur between alleles^26^. Several techniques have been developed to study promoter-enhancer interactions on a genome-wide scale. Hi-C was the founder technique to define TADs and higher-order chromatin interactions genome-wide^27^. Based on TADs and long-range interaction data it is possible to move past the prediction that the closest gene is the likely target since it is relevant to consider the genes located in the TADs where the candidate regulatory variants are located. Targeted chromosome conformation capture (Capture-C, ChiC, HiCap) is a novel technique evolved from Hi-C yielding promoter-enhancer interactions to a much higher resolution^25,28,29^. A single HiCap experiment can employ approximately 40,000 capturing probes targeting gene promoters annotated in RefSeq database to provide a genome wide promoter-enhancer interaction network.

In this study we address the three steps presenting a systems strategy to find functional gene regulatory variants, the TFs that bind differentially between alleles and novel strategies to detect the regulated genes. We identified 17,293 unique AS-SNPs across 7 lymphoblastoid cell lines. To elucidate possible regulatory mechanisms we evaluated their associations to immune GWAS traits and to gene expression in B cells. Lastly, we integrated long-range 3D interactions data to identify putative target genes and motif predictions to identify TFs whose binding may be affected by AS-SNPs.

## Results

We used ChIP-seq data and genomic sequences from seven B cell lines to search for AS-SNPs. Initially the two personal genomes were created by replacing the reference bases at variable sites with the bases supplied by a cell-specific SNPs collection (see Methods). The ChIP-seq reads from histone modifications defining promoters (H3K4me3), enhancers (H3K4me1, H3K27ac) and domain boundaries proteins (CTCF, SA.1)^30^ were then aligned to the respective genomes to identify heterozygous sites with allele specific signals and the results were corrected for genome-wide testing and pruned for false positives by filtering out AS-SNPs in CNVs and different types of repeated regions (see Methods). The cell specific collections of AS-SNPs obtained in this way were then intersected with GWAS and eQTL SNPs identified in B cells in order to link the allele-specific behavior observed in ChIP-seq to B cell specific traits or gene expression. Finally, in order to gather more information about the effect of the AS signals, we used long-range interaction data to prioritize the targets of the identified regulatory SNPs and motif analysis to identify candidate TFs mediating the regulatory process.

### AS-SNPs detection using ChIP-seq reads

**Figure 1:**
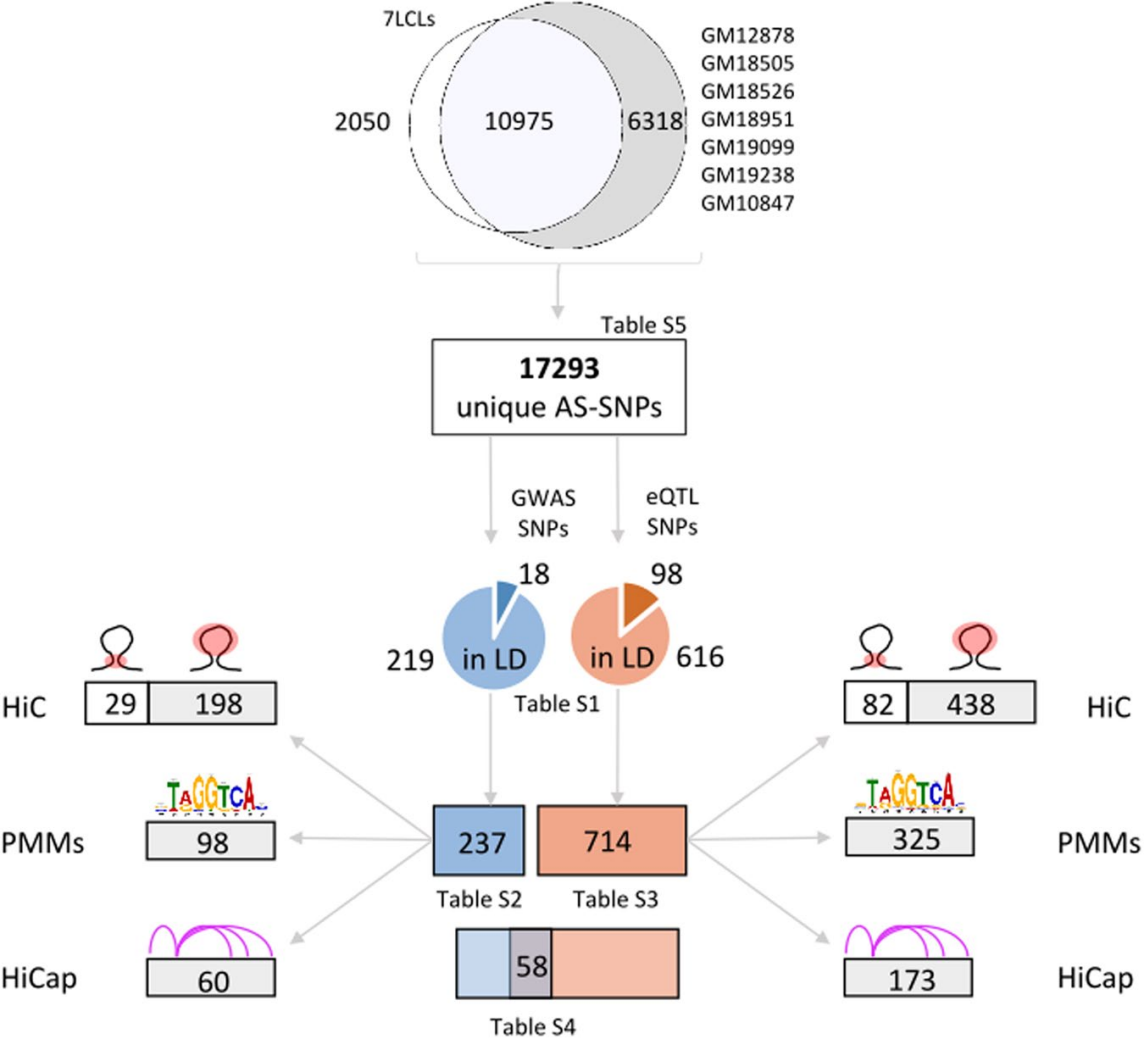
AS-SNPs identification in LCLs. 17293 unique AS-SNPs were identified running our pipeline on ChIP-seq data in 7 individual lymphoblastoid cell lines or across the cell lines (7LCLs). The collection of AS-SNPs was intersected with GWAS SNPs associated to B cell related traits and with SNPs in LD with them yielding a subset of 237 AS-SNPs associated to B cell related traits. The same approach was applied using eQTL SNPs defined in B cells and SNPs in LD with them yielding a subset of 714 AS-SNPs associated to gene expression in B cells. 58 AS-SNPs were associated to both gene expression and B cell related traits. These subsets of AS-SNPs showing evidences of biological relevance were further intersected with HiC (contact and loop domains) and HiCap data, and overlapped to TFBS predicted using PMMs.

To ensure that the AS-SNPs identified from histone modifications ChIP-seq data were due to the AS activity of the regulatory element we selected only AS-SNPs within the regulatory element whose boundaries were defined by the modified histones. To this end, we intersected the AS-SNP list with annotated elements from chromHMM and tfNet (see Methods). Across the different cell lines, we identified 17,293 unique AS-SNPs, located within annotated elements which were selected for further filtering (Fig. 1, Table 1 and Supplementary Table S5). The majority of the AS-SNPs were defined by ChIP-seq reads of enhancer specific histone modifications (33% from H3K27ac and 6,5% from H3K4me1). The promoter mark H3K4me3 defined 19% of the AS-SNPs collection while CTCF and SA.1 accounted for 5,2% and 6,9% respectively (see Supplementary Fig. S1). Finally, 29,4% of the B cell specific AS-SNPs were defined by mixed chromatin signals (e.g. H3K27ac and H3K4me1 or H3K4me3).

**Table 1:** Number of AS-SNPs identified per cell line.

Cell line	Total hz SNPs	Total AS-SNPs	Common AS-SNPs (%)	Rare AS-SNPs (%)	Population
GM12878	2221835	5318	5150 (96,8%)	168 (3,2%)	Caucasian
GM18505	2976970	2132	1936 (90,8%)	196 (9,2%)	Yoruban
GM18526	2091055	1961	1883 (96,0%)	78 (4%)	Han Chinese
GM18951	2092387	1224	1195 (97,6%)	29 (2,4%)	Japanese
GM19099	2978401	1904	1735 (91,1%)	169 (8,9%)	Yoruban
GM19238	2964106	5580	5112 (91,6%)	468 (8,4%)	Yoruban
GM10847	2560997	1457	1401 (96,1%)	56 (3,9%)	Caucasian
7LCLs		13025	11892 (91,3%)	1133 (8,7%)	
Tot. unique		17293	16094 (93%)	1199 (7%)	

**Figure 2:**
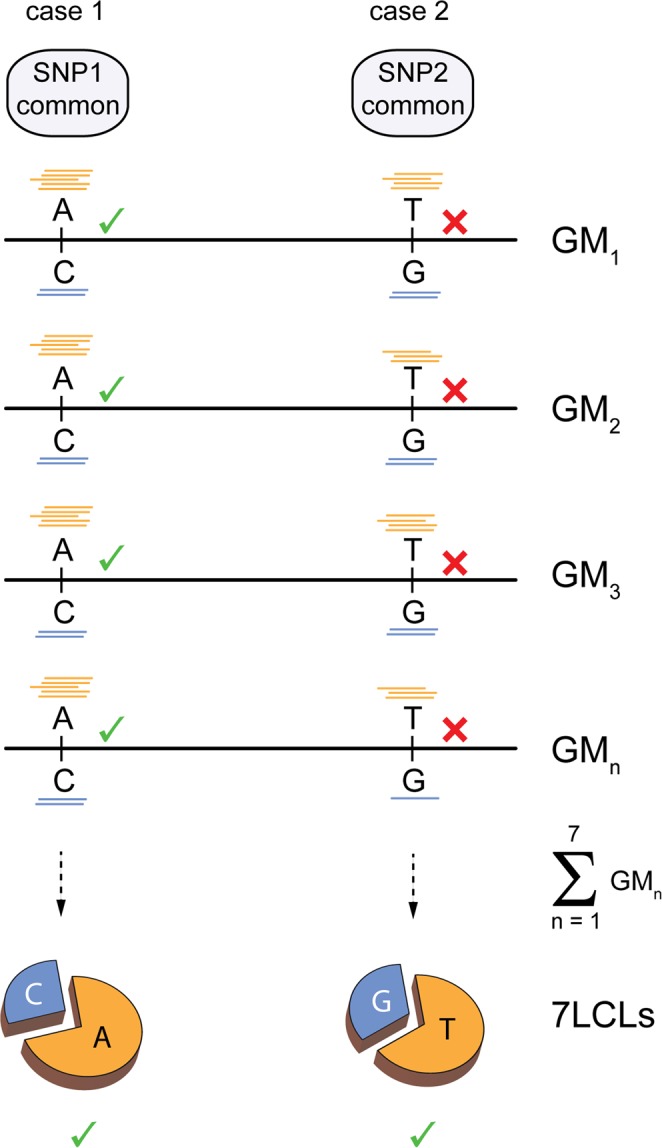
Definition of AS-SNPs at a population level. ChIP-seq reads were aligned to personalized genomes for each cell line and summed across cell lines at heterozygous positions. The resulting allele specific read count was tested for statistical significance to identify AS-SNPs defined at a population level. Case1: common AS-SNPs already observed in individual cell line maintain the statistically significant difference in reads count between the alleles also at a population level. Case2: common SNPs that are not showing statistically significant difference in read counts between the two alleles in a single cell line might surface as AS-SNPs when looking for AS binding in the population.

The number of AS-SNPs identified for each cell line was highly correlated to the number of raw ChIP-seq reads aligned to the personal genomes, since better coverage increases the chances to detect allele specific signals (see Supplementary Fig. S2). We also defined AS-SNPs across different cell lines using a cumulative approach aggregating aligned ChIP-seq reads from the 7 cell lines (7LCLs) at heterozygous positions. The rationale behind this definition of AS-SNPs at a population (n = 7) level stems from our previous observations that SNPs not showing a statistically significant difference in read counts between the two alleles in a single cell line might surface as AS-SNPs when we look for the allele specific signals in the population (Fig. 2).

We defined 13,025 unique AS-SNPs in 7LCLs (Fig. 1); 10,975 (84,3%) of them were already observed at individual cell line level (case 1 in Fig. 2) while 2,050 (15,7%) surfaced as AS-SNPs when considering the reads across the 7 cell lines (case 2 in Fig. 2). This approach is not expected to enrich the collection of AS-SNPs for previously undetected rare variants which are likely present in single cell lines.

### Associations of AS-SNPs to disease and gene expression

We wanted to identify AS-SNPs that are likely candidates to explain associations to disease in GWAS and to expression in studies of eQTLs (see Genomic Features in Methods). Therefore, the selected AS-SNPs were intersected with the SNPs with the strongest association (GWAS top hit) to selected B cell related traits from the GWAS catalog and with SNPs in high LD (r^2^ > 0.8) with GWAS top hits. Genetic control of gene activity has been analyzed in lymphoblastoid cell lines and we used a collection of eQTL SNPs (eSNPs) to investigate our AS-SNPs following the same approach as for GWAS SNPs.

Across the different cell lines we identified 237 unique AS-SNPs associated to B cell related traits and 714 unique AS-SNPs associated to gene expression (Fig. 1, and Supplementary Tables S1–S3).

18 GWAS SNPs associated to B cell related traits showed allele-specific signals while 216 AS-SNPs were identified in LD with reported GWAS SNPs. At B cell eQTL loci, 98 reported SNPs (4 in HLA region) were identified as allele specific with 603 AS-SNPs (66 in HLA region) reported in high LD. As shown in Table 1 we found several rare AS-SNPs that could contribute to disease associations. However, since they are rare they normally do not reach genome-wide significance in GWAS or eQTL studies. We found three rare AS-SNPs identified in LD with common eQTL SNPs.

We also intended to identify those that are located in the same regulatory elements as an AS-SNP that is a candidate to a GWAS or eQTL signal. We therefore intersected rare AS-SNPs with windows (±300 bp) centered at common AS-SNPs associated to traits or eQTLs. The results are reported in Supplementary Table S1: 10 and 3 rare AS-SNPs were found in ±300 bp windows centered at common AS-SNPs associated to B cell eQTLs and GWAS SNPs respectively. These rare variants are candidates to contribute to GWAS and eQTL signals and are likely to introduce heterogeneity in the studies. Overall, we identified 58 AS-SNPs (Fig. 1 and Supplementary Table S4) with strong indications of functional regulatory activity supported by association to both diseases (GWAS SNPs) and gene expression (eQTL SNPs). For these GWAS signals we have detected both a candidate regulatory variant and a predicted gene from the eQTLs.

### B cell related diseases associated to AS-SNPs

From the genetic point of view, immune system diseases represent the largest group of disease associated SNPs reported in the GWAS catalog, however these variants are in most cases the result of a statistical association and not the real functional SNPs driving the association. In this study, we identified AS-SNPs in high LD with GWAS SNPs associated with 20 unique B cell related traits/diseases (Table S8). In most cases, the identified AS-SNPs contribute to fill in the blanks to get a more complete picture of the regulatory genomic architecture at the loci flagged in GWAS.

Not surprisingly, many autoimmune disease related AS-SNPs were located in the human leukocyte antigen (HLA) region on chromosome 6. This highly polymorphic region, while essential for the adaptive immune system, has historically been challenging to study at a genomic regulatory level. Many GWAS and eQTL variants have been reported in the HLA region but the molecular mechanisms behind the associations remain elusive.

**Figure 3:**
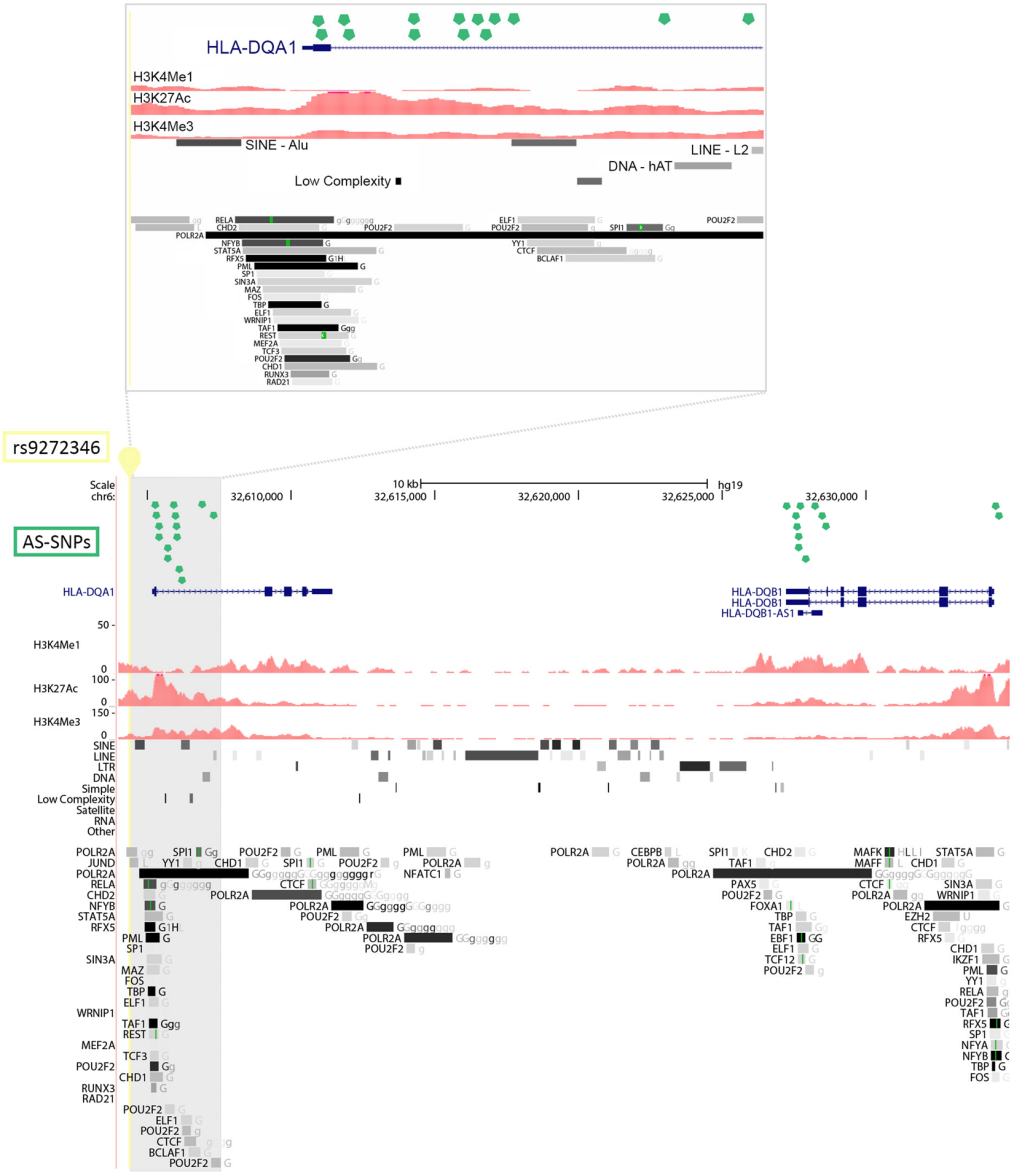
UCSC Genome browser view of the 26 AS-SNPs (green dots) in high LD with the GWAS SNP rs9272346 (yellow pin) associated to Type 1 Diabetes. Histone modifications tracks for H3K4me1, H3K4me3 and H3K27ac were retrieved from the ENCODE project for the B cell line GM12878 (scaled using vertical viewing range settings) as well as the RepeatMasker track which reports DNA sequences with interspread repeats (e.g. SINE, LINE, etc.) and low complexity DNA sequences. On the bottom track, transcription factors binding sites from ChIP-seq data from the ENCODE project for GM12878 (G) and other B cell lines (g). The insert on the top zooms into the first intron of HLA-DQA1.

One example is the GWAS SNP rs9272346 reported in the GWAS catalog as associated to T1D however, this variant is not located in a regulatory element rich in TFBS and it is located in the highly variable HLA region (Table S2 and Fig. 3). We identified 26 AS-SNPs in high LD with rs9272346 which can help to untangle the regulatory mechanism(s) at this complex region. The AS-SNPs are located in at least 10 different regulatory elements (defined by 200–300 bp in size) which show several TFBS and enhancer defining histone modifications in B cell lines. They are located in UTRs and intronic regions of the HLA alleles *DQA1* and *DQB1* which have been reported as coding for HLA epitopes most strongly associated with susceptibility for T1D^31^. Thus, our data suggest that expression varies between HLA alleles and that this may contribute to the risk of T1D and other immune diseases.

The identification of AS-SNPs at this locus illustrates the complementary power of our approach to GWAS that helps to refine the candidates for experimental validation.

Similarly, we identified AS-SNPs-harboring loci for several auto inflammatory diseases including 8 allele specific loci for CD and IBD and 11 for UC. Several AS-SNPs were also clustering at loci associated to MS (10 loci), SLE (4 loci), RA (3 loci), amyotrophic lateral sclerosis (4 loci), vitiligo (3 loci) and celiac disease (4 loci).

Outside the HLA region, in most cases, the loci defined by AS-SNPs were in close proximity with genes relevant for the disease, making them the primary targets for experimental validation of the regulatory mechanisms.

For instance, for celiac disease, we identified four loci at non HLA-genes including the AS-SNP rs2216763 located in an intron of the *RMI2* gene, important for gene stability and linked to celiac disorder^32^, two AS-SNPs (rs17035374 and rs12468278) located in two regulatory elements associated to the *PLEK* gene^33^, the AS-SNP rs6776027 in an intron of the *FRMD4B* gene, a reported celiac locus^34^ and the AS-SNP rs10511390, located in an intron of the *ARGAP31* gene involved in intestinal barrier function and linked to celiac disorder^35^.

Another example are three loci associated to RA. Two AS-SNPs, rs28157 and rs28158, were identified in the same regulatory element on chromosome 5. They are located in an intron of *C5orf30*, a reported negative regulator of tissue damage in RA^36^. Two other AS-SNPs on chromosome 6, rs28362859 and rs2233434 were located in the third intron and first exon of the *NFKBIE* gene, encoding for the nuclear factor-κB (NF-κB) inhibitor epsilon. The NF-κB pathway is well documented as active in acute inflammatory responses and in chronic inflammatory diseases like RA^37^. Finally, a locus on chromosome 9 was flagged by two more AS-SNPs, rs3761846 and rs3761847 located in an intron of *TRAF1*, coding for the tumor necrosis factor-receptor associated factor 1 and highlighting how adding more pieces of information into the regulation of the TNF pathway is crucial to better understanding this complex disease.

We also identified 61 AS-SNPs associated to “multiple traits” e.g. the AS-SNP rs11557466 identified in GM18526 and reported in Table S2, which is in LD with GWAS SNPs associated to IBD, T1D, UC, CD, RA, Primary biliary cirrhosis, SLE and Systemic sclerosis. These AS-SNPs associated to multiple traits suggest even more complex regulatory mechanisms which warrants for more detailed experimental validations and molecular interpretation. At the same time it illustrates that some functional regulatory elements and genes are shared between several immune diseases.

### Long range effects of AS-SNPs on gene regulation

**Figure 4:**
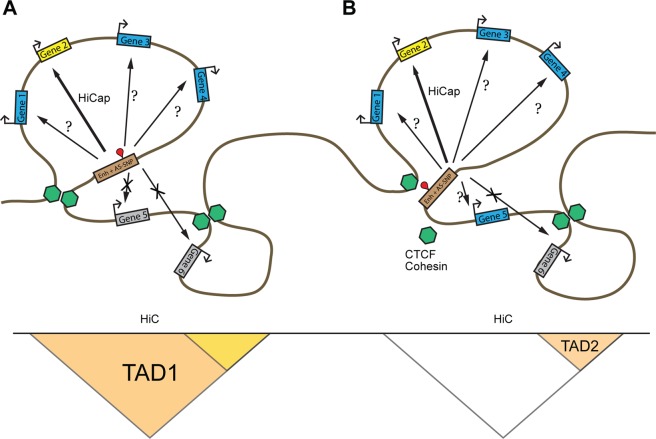
Schematic representation of using 3D interactions data to prioritize candidate target genes. (**A**) AS-SNP (red pin) in a regulatory enhancer harbored in a TAD loop defined by HiC data. The genomic architecture reduces the pool of possible target genes to genes enclosed in the TAD (genes 1–4). HiCap analyses allow narrowing the list of putative target genes even further evaluating specific probe interactions, pointing in this example to gene 2 as the likely target for the enhancer element. (**B**) The presence of an AS-SNP in a TAD interaction domain may lead to an altered assembly of the TAD formation protein complex (e.g. CTCF, cohesin, etc. represented in green) resulting in a different TAD architecture. In this example the disruption of TAD1 extends the list of putative target genes to genes 1–5.

In this study we identified AS-SNPs defined by ChIP-seq reads from CTCF and SA.1, two architectural protein involved in the topological organization of the genome. SA.1 is part of the cohesin complex, a ring-shaped protein complex which is central in the 3D organization of the chromatin. DNA loops are “extruded” through the cohesin ring to form TADs and the TADs boundaries are marked by the transcription factor CTCF, whose binding motifs oriented in divergent directions acts as “anchor” points for cohesin ring. The sites at which CTCF binds are called interaction domains and the sequences in between form a loop (TAD loop). Most interactions between promoters and enhancers happen within these loops (Fig. 4). We used publicly available HiC data from GM12878 to identify AS-SNPs located in TAD loops or TAD interaction domains. HiC data from GM12878 should be representative for interactions also in B cell lines from the other individuals in this study. We identified 4444 AS-SNPs defined by CTCF ChIP-seq reads out of the total collection of 17293 AS-SNPs. 1343 of these CTCF defined AS-SNPs overlap CTCF motifs predicted using PMMs. When the variants are located in the TAD interacting domains they are likely to influence the binding of factors involved in the TAD formation (Fig. 4B). This could alter the interaction and consequently the loop structure. Across the cell lines, we found 82 and 29 unique AS-SNPs located in TAD interacting domains and associated to gene expression and B cell related traits respectively. This may indicates that loop structures may be polymorphic in the population. We also found 438 unique AS-SNPs associated to gene expression and 198 to B cell related traits harbored in annotated TAD loops in GM12878 (Fig. 1 and Supplementary Tables S2,S3). Each of these AS-SNPs is likely to regulate the expression of a gene in each TAD, however the target gene(s) cannot be inferred from the HiC data alone.

HiCap is a modification of HiC in which the ligated DNA molecules are hybridized to an array of candidate regulatory elements such as promoters of all genes and/or selected enhancers. We utilized in-house HiCap data in GM12878 generated with probes placed at the promoter regions of genes to identify all the distal elements interacting with a promoter and in particular regulatory elements with AS-SNPs. We found 173 unique AS-SNPs associated to gene expression and 60 to B cell related traits in distal regulatory elements that interact with defined promoters. This points to specific genes likely regulated differently between alleles. We also designed HiCap probes located in regulatory elements with AS-SNPs to detect interactions with promoters or other enhancers. In this more limited data set we found six additional AS-SNPs associated to gene expression and two to B cell related traits in distal elements that form 3D interactions with promoters.

### Well-annotated candidate regulatory variants

**Figure 5:**
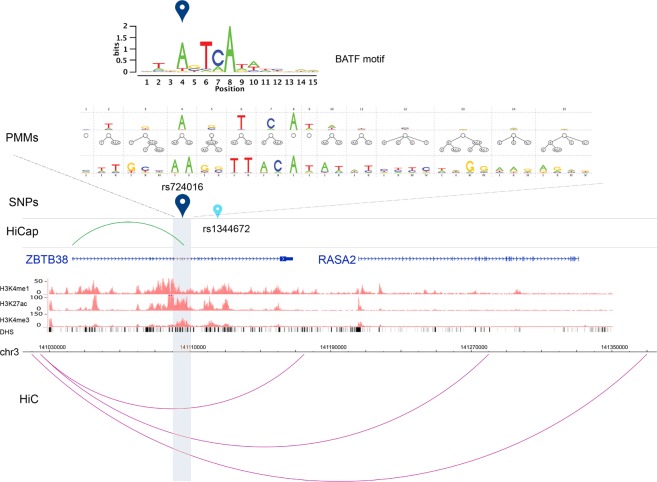
Multi-layered evidences for a candidate functional AS-SNP in LD with an eQTL SNP. AS-SNP rs724016 is in LD with an eQTL SNP (rs1344672) associated to the expression of *ZBTB38*. The SNPs are located in a genomic region with a multi loop TAD architecture defined by HiC data (purple interactions) that narrows the possible target genes to two candidates: *ZBTB38* and *RASA2*. HiCap data (green interaction) show that the probe harboring rs724016 interacts with the promoter of *ZBTB38* confirming the association observed in the eQTL study. The distal probe region (~5 kb) is highlighted in gray. Histone modifications tracks for H3K4me1, H3K4me3 and H3K27ac were retrieved from the ENCODE project for the B cell line GM12878 (scaled using vertical viewing range settings) as well as the DNaseI hypersensitive clusters (DHS). The PMM tracks show the networks of intra-motif dependencies at each position using PMMs with defined parameters. The sequence logos for the TF binding motif of BATF has been computed from putative BSs predicted by PMMs.

**Figure 6:**
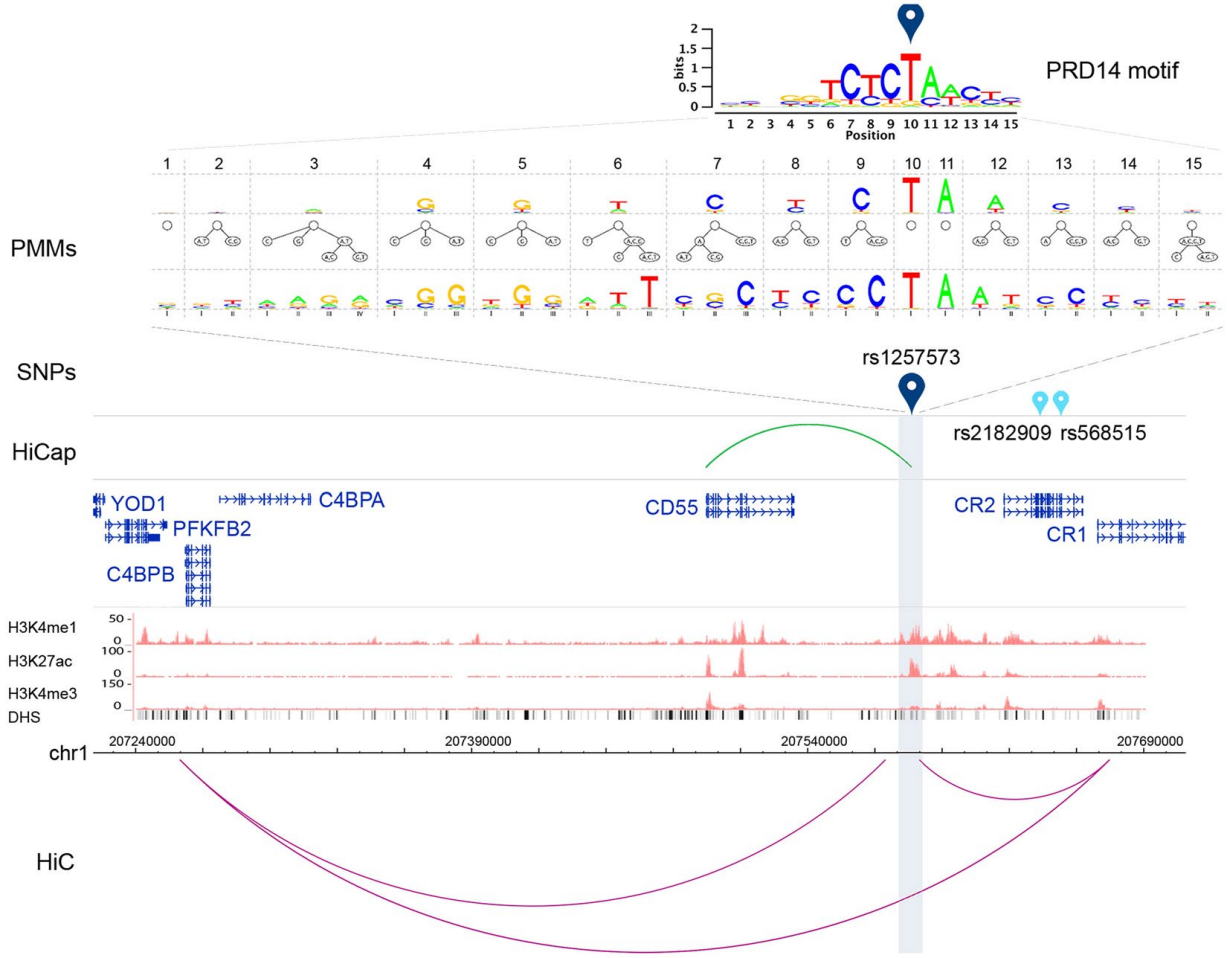
Multi-layered evidence for candidate functional AS-SNPs in LD with two eQTL SNPs. AS-SNP rs1257573 is in LD with two eQTL SNPs (rs2182909 and rs568515) associated to the expression of *CR2*. The SNPs are located in a genomic region with a multi loop TAD architecture defined by HiC data (purple interactions) that narrows the possible target genes to few candidates. HiCap data (green interaction) showed an interaction between the promoter of *CD55* and the regulatory element harboring the AS-SNP rs1257573. The distal probe region (~5 kb) is highlighted in gray. Histone modifications tracks for H3K4me1, H3K4me3 and H3K27ac were retrieved from the ENCODE project for the B cell line GM12878 (scaled using vertical viewing range settings) as well as the DNaseI hypersensitive clusters (DHS).The PMM tracks show the networks of intra-motif dependencies at each position using PMMs with defined parameters. The sequence logo for the TF binding motif of PRD14 has been computed from putative BSs predicted by PMMs.

HiCap data revealed 173 AS-SNPs associated to gene expression and 60 to B cell related traits showing promising evidence of regulatory effect supported by multiple data sets. In 79 cases of AS-SNPs in LD with eQTLs, HiCap analyses confirmed the distal interactions of regulatory elements harboring the AS-SNPs with the genes identified in the eQTL studies. One example (Fig. 5) is the AS-SNP rs724016 in LD with the eQTL SNP rs1344672 that was associated to the expression of *ZBTB38*. rs724016 is located in a multi loop TAD defined by HiC data. The TAD spans ~0.3 Mb and contains two different genes (*RASA2* and *ZBTB38*). HiCap data show that the probe harboring rs724016 interacts with the promoter of *ZBTB38* confirming the association observed in the eQTL study. This highlights the screening power of long-range interaction data in defining the target(s) of regulatory elements. Motif analysis (see below) showed also how rs724016 altered binding sites for two TFs, BATF and NDF2, suggesting a putative molecular mechanism for the regulatory activity. In another 94 cases, HiCap data detected an interaction with a target gene different from the one detected in eQTL studies suggesting how HiCap can help establish the most likely target gene. An example is the AS-SNP rs1257573 (Fig. 6) predicted to affect a binding site for the transcription factor PRD14 and located in a ~0.4 Mb TAD on chromosome 1 in a loop harboring several different genes. rs1257573 is in LD with two eQTL SNPs (rs2182909, rs568515) associated to *CR2* expression. HiCap data showed how the distal element with rs1257573 is interacting with the promoter of the *CD55* gene located in the same TAD loop. In this case the eQTL might be driven by another SNP with a lower LD than the threshold used in this study.

We also used the data to prioritize gene targets for AS-SNP associated to diseases via GWAS. An example is two AS-SNPs, rs11811536 and rs12032504 (Supplementary Fig. S3), located in two regulatory elements ~2 kb apart on chromosome 1 that are in LD with the GWAS SNP rs10911390 associated to Systemic lupus erythematosus (SLE). The GWAS SNP is located in a region sparsely annotated with TFBS ~12 kb downstream from these two putative regulatory elements, suggesting that the GWAS SNP is not the real driver of the association to SLE. All the SNPs are located in a TAD domain with a complex looping architecture spanning ~0.4 Mb and enclosing 5 genes as defined by HiC data. Our HiCap data suggest that the regulatory elements harboring the two AS-SNPs interact with the promoters of two of the 5 genes: *APOBEC4* and *RGL1*, which have both been associated to SLE susceptibility loci following meta-analysis with replication studies^38^. Motif analysis suggested that rs11811536 could alter the binding of TFs RUNX3 and STA5B and rs12032504 of TFs NDF2 and Z280D. The complexity of the regulatory mechanism of this regulatory locus on chromosome 1 is highlighted by several other GWAS SNP that have been reported in association to SLE in different populations: Gateva *et al*.^39^ reported rs10911363 as associated with SLE in Caucasians, Alarcon-Riquelme *et al*.^40^ reported rs13306575 as associated with SLE in Native Americans, and Bentham *et al*.^38^ reported rs17849501 as associated with SLE in Europeans. rs17849501 was further reported by Morris *et al*.^41^ as associated to SLE in Europeans but not in Chinese individuals. The AS-SNPs we identified in this study are in LD with rs10911390 reportedly associated to SLE in Asians and are likely to help explaining the association in this population but other variants may explain the associations in other populations. Overall, our approach not only points to the SNPs that are likely to drive the association observed in GWAS but it could also provide a possible direct genetic evidence of the target gene(s) and suggest TFs with differential binding.

### Effect of AS-SNPs on TF binding

PMMs were built for 392 TFs and were used to scan genomic regions for BSs centered at the identified 17,293 AS-SNPs ± 300 bp. A total of ~1.6 million BSs were identified (p-value < 1 × 10^−3^). Putatively altered TF-BSs were predicted for 98 (41.3%) and 325 (45.5%) of the AS-SNPs associated to GWAS or eQTLs respectively (Fig. 1).

The Jaccard similarity coefficient measured between the collection of AS-SNPs and identified BSs was calculated as a measure of the fraction of the AS-SNP collection which affect a specific TF binding site. We also determined the fraction of predicted BSs that is altered by AS-SNPs for each TFs. The BS predictions allow also defining a gain or loss in the binding due to the presence of AS-SNPs based on the affinity score calculated for the two alleles.

Supplementary Table S7 shows the results of the motif analysis for AS-SNPs and AS-SNPs associated to GWAS and eQTLs SNPs: some TFs (e.g. BLC3, BMAL2, HDX1) show a fraction of up to 20% of altered BSs suggesting a possible enrichment of AS-SNPs specifically modulating the binding of these TFs. Other TFs display a smaller fraction of altered BSs but with a more dramatic effect on the binding score with a gain (e.g. HXC13, ZN274, REST) or loss (e.g. MEF2A, EVX2, GLIS3, NCOA3) of binding affinity due to the presence of AS-SNPs. In general, a loss of binding scores is more prominent among the predicted BSs overlapping AS-SNPs where we observe a score loss for ~54% of the predicted BSs. This pattern may also be observed with regard to the individual PMMs, where ~77% of the PMMs show a loss for more than 50% of their predicted BSs. A similar trend was observed also in subsets of AS-SNPs associated to GWAS or eQTLs SNPs.

## Discussion

The nature of the AS signals obtained using raw reads from ChIP-seq experiments of histone modification is qualitatively different from the signals from ChIP of TFs. An AS signal derived from ChIP-seq data of a TF reflects the preferential binding of the specific TF studied to one or the other allele at specific heterozygous sites. This concept relies on the rather straightforward idea that a functional SNP can alter the TFBS which, in turn, will result in a unbalanced representation of ChIP-seq reads favoring one of the alleles (Supplementary Fig. S4a). Histone modifications, on the other hand, represent markers of activation (or repression) happening at promoters and enhancers. AS signals observed from ChIP-seq of histone modifications are likely to reflect the activity of the regulatory element defined at its boundaries by the modified histones (Supplementary Fig. S4c). It is worth to remind that enzymes that introduce histone modifications (e.g. (de)metylators and (de)acetylators) do not have sequence specificity which could be altered by variants as described above. As a result, the difference in read counts from histone modifications ChIP-seq data observed at heterozygous positions points out the allele that was more active due to the action of a TF that bound to the regulatory element.

The choice to focus on histone modifications H3K4me1 and H3K27Ac, which mark enhancer regions, stems from the observation that most of the gene regulation happen in non-coding part of the genome and mostly not in promoters. In addition, the majority of the reported disease associated variants in the GWAS catalog maps to the non-coding part of the genome. Furthermore, it has been shown that multiple enhancers can regulate the same gene and that a promoter of a gene can act as an enhancer element for another gene so we selected H3K4me3 to improve the pool and the numeric density of raw ChIP-seq reads and boosting the chances to pinpoint regulatory elements. Distal enhancers are largely tissue specific and in line with expectations, we found that most AS-SNPs where detected by enhancer marks, sometimes with a combined signal from H3K4me3.

We confirmed the direct correlation between read number and read depth in the identification of AS-SNPs. The GM12878 and GM19238 cell lines had several ChIP-seq replicates that provided even further coverage to detect AS signals at a statistically significant level and showed the highest number of AS-SNPs. In our previous collections of AS-SNPs based on TF ChIP-seq data^6^ we observed 13–19% of rare variants whereas here we detect 7% on average. We observed a greater proportion of rare variants in the cell lines derived from individuals of African descent, which is in agreement with the greater amount of genetic variation observed in African populations. In the present study we used reads of 101 bp which are easier to map correctly leading to a lower fraction of false positives. The data could also be influenced by the quality of the SNP calling in the respective cell lines. Nevertheless, using input data of high quality we find that 7% of candidate functional variants are rare in the population. Several types of data have shown that rare variants also have larger effect sizes^42^. We therefore predict that rare sequence variants may explain a larger fraction of expression variation than what was recognized in the past. They may also explain why GWAS and eQTL studies in different populations find associations to different common variants. Rare variants differ between populations and in one they may tag one common variant whereas a second population may have other rare variants that are tagged by another common SNP. In this study we find 16 rare AS-SNPs that could contribute to such processes.

The intersection of B cell AS-SNPs collections with GWAS and eQTL SNPs is aimed at adding a biological relevance layer. As observed before, the associations reported in GWAS and expression studies were directly supported by allele specific signals in 10% of the cases on average. The vast majority of the identified AS-SNPs were in LD with reported associated SNPs and likely to be the regulatory variants driving the associations. Rare AS-SNPs were not expected to overlap previously reported SNPs associated to disease and gene expression due to the study design that preclude rare variants from being statistically significant. Rare variants identified in LD with eQTL SNPs or in ±300 bp regions around associated SNPs are candidates to be relevant in the regulatory activity of the enhancers where they are located.

We used publicly available 3D interaction data to prioritize the candidate target genes for the regulatory elements. TAD definitions were applied to restrict the pool of possible target genes based on the assumption that AS-SNPs in regulatory elements within a TAD loop are likely to regulate genes harbored in the loop. This approach is qualitatively superior to an association by “proximity” historically used to link trait-associated GWAS SNPs to genes. The presence of AS-SNPs in the TADs interacting domains is also of particular interest since variations could disrupt the architecture of the TAD and allow interactions into neighboring TADs. The TAD domain delineates the number of genes that could be regulated by an AS-SNP but does not yet provide a direct evidence of the interaction. We used in house HiCap data produced in GM12878 cell line to retrieve long range promoter-enhancer interactions to add another layer of precision in the ranking of the candidate regulatory AS-SNPs. HiCap data allowed us to refine the gene target predictions for 173 AS-SNPs associated to B cell eQTLs: in 79 cases the gene expression associations observed in the eQTLs studies was confirmed while in 94 cases HiCap data suggested a different target gene(s). It is crucial to keep in mind that the architectural regulation of gene expression might be more complex than the association reported by eQTL studies and that integrating long-range interactions data should be considered as a complementary approach when using eQTL data to inform GWAS signals on possible target genes.

In order to identify the TF(s) whose binding could be altered by the presence of AS-SNPs we performed motif analyses using PMMs that take into account intra-motif dependencies. The idea is to evaluate whether BSs for specific TFs are overrepresented at regulatory elements harbouring AS-SNPs. Applying this kind of analysis to subsets of AS-SNPs associated to specific diseases via LD with GWAS SNPs could possibly define sets of TFs relevant for the disease pathology.

These predictions, however, depend on (i) the choice of the significance-based threshold affecting the number of predicted BSs, (ii) the size of the genomic regions around SNPs considered, and (iii) the selection of TFs for which PMMs are built. Moreover, since the number available ChIP-seq data for TFs required for learning motif models is still rather limited, current predictions likely underestimate the number of BSs affected by AS-SNPs.

In conclusion, we presented a systems strategy to find functional gene regulatory variants, the TFs that bind differentially between alleles, and novel strategies to detect the regulated genes. The collection of AS-SNPs presented here offers a set of promising candidate regulatory variants supported by several layers of evidence to prioritize experimental validations aimed at improving the knowledge of the molecular mechanisms of many immune-mediated diseases.

## Materials and Methods

### ChIP-seq data

We used publicly available ChIP-seq data generated in 7 lymphoblastoids cell lines (GM10847, GM12878, GM18505, GM18526, GM18951, GM19099 and GM19238) for three histone modifications: H3K27ac, H3K4me1 and H4K4me3 and two genome architectural proteins: CTCF and SA.1^30^. The data sets were downloaded in short read archive format (.sra) from Gene Expression Omnibus (accession no. GSE50893). The reads quality was assessed using Phred64/33 scores with a quality cutoff requirement of 20.

### Creation of diploid genomes

We use the diploid genome reconstruction module from the ALEA toolbox^11^ that takes a list of phased variants and a reference genome as inputs. We utilized the 1000 Genomes phase 3 phased genotypes for variants called in the selected 7 cell lines. (ALL.chr?.phase3_shapeit2_mvncall_integrated_v5a.20130502.genotypes.vcf.gz) and the Genome Reference Consortium Human genome build 37 (GRCh37) as the backbone reference to build two *in silico* personal genomes for each cell line. ChIP-seq reads aligning to the reference and alternative genomes are referred to as G1 and G2 in Supplementary Tables S2–S5.

### AS-SNPs definition

The AS-SNPs discovery was adapted from our established pipeline^6^ which was completely rewritten in Perl and is available on http://bioinf.icm.uu.se/repositories.php. The pipeline is composed of 5 modules, each handling one or more aspects of the process. The division into separate modules is aimed at lower the computational cost. All settings and controls are handled via a single configuration across modules (see Extended Methods in Supplementary Information for more details).

### Genomic Features

AS-SNP collections were intersected and filtered with several publicly available databases:

- GWAS SNPs associated to selected B cell related traits (Table S8)^6^ from the NHGRI GWAS catalog^43^. A total of 1545 unique GWAS SNPs were retrieved plus 26300 SNPs in high LD (r^2^ > 0.8) with them.
- Collections of eQTL SNPs associated to gene expression in lymphoblastoid cell lines^44^. A total of 5565 unique eQTL SNPs were retrieved from the GEUVADIS databse (https://www.ebi.ac.uk/arrayexpress/files/E-GEUV-1/analysis_results/) plus 66935 SNPs in high LD (r^2^ > 0.8) with them.
- 1000 Genomes SNP collection (1000 Genomes project, phase3-shapeit2-mvncall-integrated-v5a.20130502)
- List of signal artifact blacklisted ENCODE regions^45^, centromeric and telomeric regions and GM12878 CNVs
- RegulomeDB^46^
- ChromHMM^47^/tfNet^48^. Annotated regions were collected from tfNet where chromatin states were condensed into 7 categories: enhancer, heterochromatin, insulator, mixed, promoter, repressed and transcribed. AS-SNPs were selected when located into enhancers, promoters, mixed or insulators regions.
- DNase I hypersensitive sites (DHSs) in GM12878 were downloaded from the Gene Expression Omnibus (GEO): study accession code is GSM816665, ENCODE file accession code: ENCFF001UVC.

### 3D Long-range Interactions

Previously, candidate target genes were selected by proximity with the GWAS SNPs but recent advances in studies of long-range 3D chromosomal interactions now offer new tools to detect direct evidence of interaction between promoters and their distal regulatory elements. Such experiments are done in cells or tissues that were fixed and chromatin fragmented using a restriction enzyme and the DNA ends ligated under extremely diluted conditions to create intra- rather than inter-molecule ligations. The DNA is then processed for paired-end sequencing and the reads were aligned to establish which ones were derived from promoter and enhancer sequences, thereby establishing long-range interactions.

A probe set containing 38,059 probes was used to capture Hi-C-GM12878 material (Supplementary Information and Supplementary Table S6A,B). Of those, 35,924 probes target 19,875 annotated RefSeq promoters. 878 regions with no annotation or regulatory potential were targeted with 1,412 probes. 462 SNPs that showed allele-specific ChIP-peak loading were targeted with 723 probes. The experiments were performed in three replicates and the average capture rate was around 60%. The raw reads were processed using HiCUP (Hi-C User Pipeline)^49^ to remove invalid and duplicate pairs (see Supplementary Table S6B for library quality metrics). The valid pairs were then analysed using HiCapTools to call significant probe-anchored interactions^50^. Proximities of probes with no known annotation or regulatory potential were used to obtain background contact frequencies. They were then employed to calculate p-values for each proximity anchored at promoter or SNP probes.

Interacting pairs that were supported by at least three reads and passed the significant threshold (p-value < 0.05) in at least two of the replicates were retained. In total 130,915 interacting anchor loci were obtained. Distal interactions were defined as regions that interacted with the designed probes (promoters) or other distal genomic regions. Therefore, the final set contained regions that directly interacted with the designed probes (direct interactions) and regions that indirectly interacted with the designed probes (indirect interactions) through one or more distal regions (known as “hops”). AS-SNPs that overlapped the interacting regions (promoter/distal) were further analysed.

Additionally, we obtained Topologically Associated Domains (TADs) for GM12878 from Rao *et al*.^51^. For each TAD, contacting domains were defined as the two interacting anchor points. Loop domains were defined from the end point of the first contact domain to the start point of the second contacting domain. Genes that were located within each loop domain were assigned to the corresponding loop. Loops that contained the same sets of genes were unified to remove duplicates. Additionally, genes that overlapped the contacting domains were assigned as the anchor genes for the overlapping contacting domain.

### Motif analysis

For building a large collection of PMMs^52^, we joined all training and test data sets (separately for each TF) from the HOCOMOCO database^17^ (http://hocomoco.autosome.ru/downloads, accessed December 5, 2016) and learned PMMs by the *de novo* module of the InMoDe software^52^ using default parameters, yielding a total of 404 PMM models for 392 TFs. Six TFs (IRX3, NKX22, SP3, TBX3, TF65, and ZBED4) did not yield a single prediction on the input files for the given p-value threshold and have not been included in the analyses.

We extracted the genomic sequences surrounding bi-allelic AS-SNPs in a window of 300 bp up- and downstream from the polymorphic site. The choice of 300 bp windows was driven by our previous work (Diamanti K *et al*.)^48^ aimed at identifying putative regulatory regions based on ENCODE data. We observed that when the distance threshold between consecutive (ChIP- or DNase-seq) peak summits was set to 300 bp it was greater than the distance between 87% of the input TFBS data essentially suggesting that the majority of the regulatory regions identified were ~300 bp in length. For this we used the Bioconductor packages GenomicRanges^53^ and BSgenome.Hsapiens.UCSC.hg19 (The Bioconductor Dev Team, 2014) and output for each AS-SNP two 601 bp sequences, one centered at the reference allele and the second at the alternative allele of the AS-SNP.

These sequences were then scanned for motif occurrences in a sliding window approach. For the motif scores, p-values were computed per motif by (i) di-nucleotide shuffling of all input sequences, (ii) computing all scores on the shuffled sequences, (iii) fitting a Gaussian density to the distribution of scores, and iv) determining the p-value of a given motif score from this Gaussian distribution. Predictions were filtered by a p-value of at most 1E^−3^.

For each input sequence with a prediction above the threshold for at least one of the variants, we collected the motif scores and associated p-values for both variants. For the following analyses, we only considered binding site predictions that are affected by a specific SNP, i.e., that overlap a SNP and, hence, show a difference in motif score and p-value.

## Supplementary information

Supplementary Information

Table S1

Table S2

Table S3

Table S4

Table S5

Table S6

Table S7

Table S8

## Data Availability

ChIP-seq data sets for the 7 LCLs were downloaded from Gene Expression Omnibus (accession no. GSE50893). AS-SNPs collections were intersected with several publicly available databases listed in the Genomic Feature section. The bioinformatics pipeline modules can be accessed from: http://bioinf.icm.uu.se/repositories.php HiCap data generated in GM12878 have been submitted to ENA (https://www.ebi.ac.uk/ena), primary accession number: PRJEB25327.
