# Supplementary material for: Allele specific chromatin signals, 3D interactions, and motif predictions for immune and B cell related diseases
Source: Sci Rep. 2019 Feb 25;9:2695. doi: 10.1038/s41598-019-39633-0 (PMC6389883; doi:10.1038/s41598-019-39633-0)
Supplement: Supplementary file 1 — Supplementary Information [file 41598_2019_39633_MOESM1_ESM.pdf]

Supplementary Information

Allele specific chromatin signals, 3D interactions, and motif predictions for immune and B cell related diseases.

Marco Cavalli, Nicholas Baltzer, Husen Umer, Jan Grau, Ioana Lemnian, Gang Pan, Ola Wallerman, Rapolas Spalinskas, Pelin Sahlen, Ivo Grosse, Jan Komorowski and Claes Wadelius

Extended Methods

AS-SNPs definition.

The bioinformatics pipeline to define AS-SNPs from ChIP-seq data has been developed from our original design (Cavalli et al.Hum Genet 2016) with the inclusion of new filtering steps designed to handle ChIP-seq data for histone modifications, more stringent quality controls and minimizing the computational costs. The bioinformatics pipeline modules can be accessed from: http://bioinf.icm.uu.se/repositories.php

Here we report the main steps:

1. Relevant files for each factor are downloaded from GEO (accession no. GSE50893).

2. ChIP-seq reads from .sra files are converted to .fastq with fastq-dump (SRA Toolkit).

3. fastq files for pair ends reads (202 bp) are split into single reads of 101 bp.

4. Reads are aligned using bowtie2 (http://www.bioinformatics.babraham.ac.uk/projects/ASAP/) against a reference (GRCh37) and an alternative genome built using the ALEA toolbox. The reference and alternative genomes are referred to as G1 and G2 respectively in Additional files 2-5.

5. The aligned reads for each genome are filtered using Phred33/64 at q20.

6. The reads are counted for each allele at each heterozygous position (hzSNPs) for the two genomes.

6b. Reads are also counted across 7 different cell lines summing up the read counts at heterozygous (hz) positions (7LCLs)

7. Only hzSNPs with reads aligning on both alleles are retained.

8. Each hzSNP is filtered by region-maps from ChromHMM and tfNet. The retained hzSNPs fall in putative regions associated with enhancers, promoters, insulators regions, or a mix of these.

9. hzSNPs located in centromeres, telomeres and CNVs defined in GM12878 are excluded.

10. A binomial test is applied to determine hzSNPs which show a statistical significant difference between reads aligned to each allele, and the results were corrected for multiple testing with the Benjamini-Hochberg algorithm.

11. All hzSNPs with a binomial test p-value above 0.05 are removed together with hzSNPs with fewer than 10 reads on either allele.

12. The remaining hzSNPs, called AS-SNPs, are intersected with SNPs from the 1000 genomes project in order to retrieve the allele frequency (AF) for AS-SNPs and define common (AF $\geq$ 0.01) or rare (AF <0.01) AS-SNPs.

13. AS-SNPs in ENCODE blacklisted regions are removed.

14. AS- SNPs are assigned their RegulomeDB scores to help in the prioritization of putative functional variants.

15. Common and rare AS-SNPs are intersected with collections of eQTL and GWAS SNPs B cell specific and SNPs in high LD ($r2 > 0.8$) with these.

16. The rare AS-SNPs are also intersected with genomic windows of 300 bp around common AS-SNPs. Rare AS-SNPs harbored in the same elements of common AS-SNPs are labelled 'extended' SNPs (see Table 2 in main text).

After the computations are completed, there are multiple scripts to describe the final results for each table. Results can be summarized in a various table formats via the reporting feature that can generate Excel tables with allow to intersect collections of AS-SNPs with data from any other study providing a .bed formatting.

High-throughput chromosome conformation capture with targeted sequence capture (HiCap)

Lymphoblastoid cells GM12878 were grown in a controlled environment of 37°C under 5% of carbon dioxide in RPMI-1640 growing medium with 2mM L-glutamine and 15% of fetal bovine serum. A blend of penicillin and streptomycin was used to prevent bacterial contamination.

High-throughput chromosome conformation capture followed by targeted sequence capture (HiCap) was performed on cross-linked GM12878 nuclei pellets as detailed below. The experiments were carried out in three biological replicates with approximate number of 5 million cells per experiment. The cells were cross-linked with 1% of formaldehyde for 10 minutes followed by cell lysis and nuclei isolation. The chromatin was then solubilized by sodium dodecyl sulfate (SDS) dilution and enzymatically digested with 1µL/µg of *fast digest Mbo*I (↓GATC; Thermo Fisher Scientific) for 4.5 hours at 37°C. SDS was quenched with Triton X-100 before the enzymatic reaction. Biotin labeling of digested DNA ends was employed for later enrichment of the aimed ligation product. A mix containing biotin-14-dATP with the reaction catalyzed by Klenow fragment of DNA Polymerase I filled the protruding 5' overhangs created by the restriction enzyme. The enzymatic activities were quenched by a brief incubation at 75°C in a presence of 10mM of EDTA. Consequently the material

was ligated with 12 Weiss units of T4 DNA ligase (New England Biolabs) for 4.5 hours at 16°C favoring intra-molecular blunt end ligation of cross-linked fragments. The ligation product was then purified using phenol-chloroform-isoamyl alcohol (25:24:1) followed by precipitation with sodium acetate pH 5.2 and absolute ethanol. RNA contamination was removed by RNase A treatment for 1 hour at 37°C. Subsequently the purified chimeric DNA was treated with T4 DNA Polymerase to remove the unligated ends containing biotin mark (reaction artifacts). Resulting product was then sheared for 6 cycles of 60 seconds using the sonication system by Covaris Inc. with these settings: duty cycle of 10%, intensity of 5, and cycles per burst of 200. The sonicated fragments were then used to prepare DNA libraries using KAPA HTP Library Preparation kit for Illumina. Platforms following manufacturer’s protocol entailing the end-repair of the amplified fragments, as well as A-tailing and TruSeq LT (Illumina Inc.) adapter ligation with the addition of biotin-avidin enrichment of the DNA fragments step before the adapter ligation. Subsequently the DNA libraries underwent another enrichment step using a part of the kit SureSelect XT Target Enrichment System for Illumina Paired-End Multiplexed Sequencing libraries (Agilent). In this step stringent hybridization of pre-designed custom RNA oligonucleotide probes was performed to capture the libraries with targeted sequences. The protocol employed blocking the Illumina adapter sequences with xGen Universal blocking oligonucleotides (Integrated DNA Technologies) followed by custom probe hybridization of 24 hours and stringent wash of captured DNA libraries. The resulting enriched DNA libraries were then in-house sequenced via Illumina single index, paired end sequencing using NextSeq 500 system (Illumina Inc.).

Qubit fluorometric quantitation (Invitrogen) and 2100 Bioanalyzer system (Agilent) were used for DNA quality and quantity controls throughout the protocol.

Supplementary Figure S1

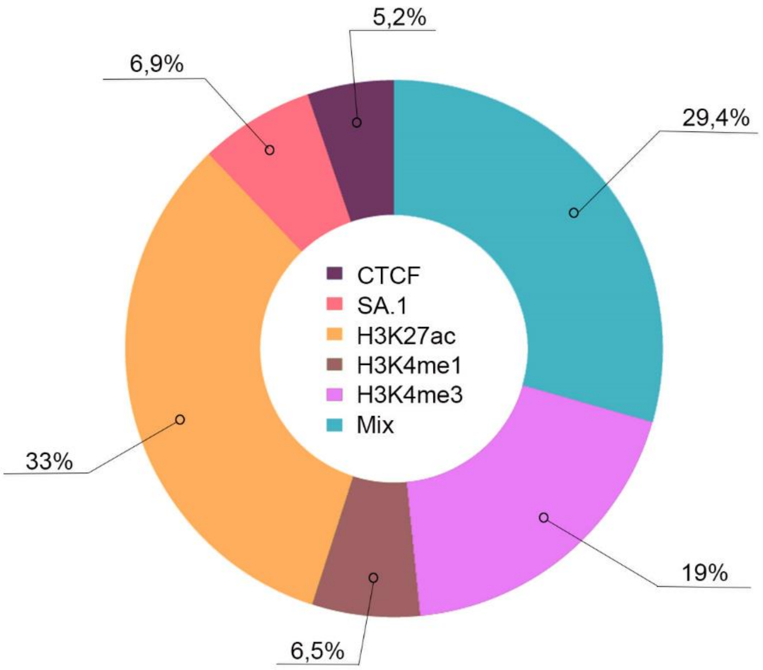

Figure. S1 AS-SNPs defined by ChIP-seq reads from histone modifications defining promoters (H3K4me3), enhancers (H3K4me1, H3K27ac), domain boundaries proteins (CTCF, SA.1) or a combination of signals (Mix).

Supplementary Figure S2

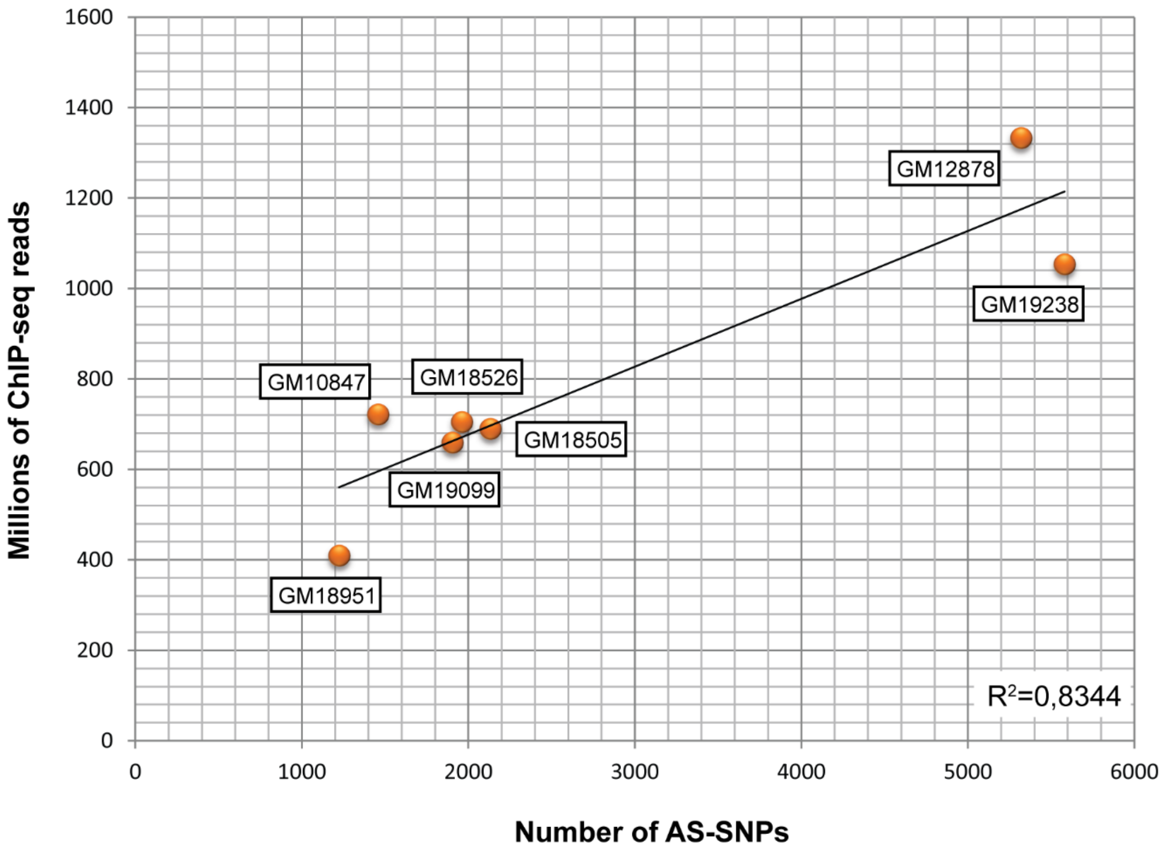

Figure. S2 Correlation between number of ChIP-seq reads and AS-SNPs detected for each cell line

Supplementary Figure S3

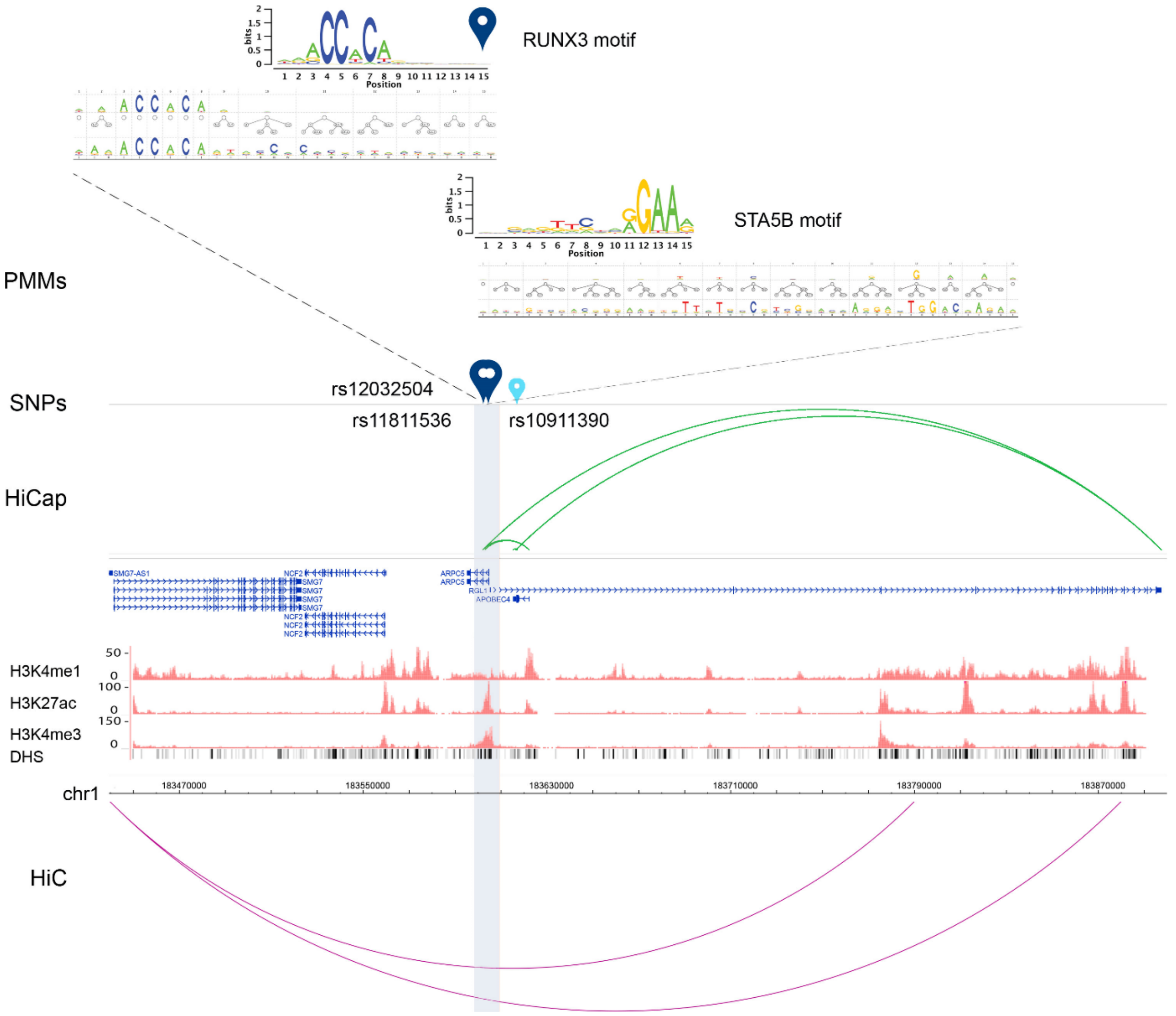

Figure. S3 Multi-layered evidences for a candidate functional AS-SNP in LD with a GWAS SNP. AS-SNP rs12032504 and rs11811536 are in LD with the GWAS SNPs rs10911390 associated to Systemic lupus erythematosus (SLE). The AS-SNPs are located ~2kb apart in a genomic region with a multi loop TAD architecture defined by HiC data (purple interactions) that narrows the possible target genes to five candidates: *NCF2*, *SMG7*, *ARPC5*, *APOBEC4* and *RGL1*. HiCap data (green interactions) suggest that the regulatory elements harboring the two AS-SNPs interact with the promoters of two of the 5 genes: *APOBEC4* and *RGL1*. The distal probe region (~5kb) is highlighted in gray. Histone modifications tracks for H3K4me1, H3K4me3 and H3K27ac were retrieved from the ENCODE project for the B cell line GM12878 (scaled using vertical viewing range settings) as well as the DNaseI hypersensitive clusters (DHS). The sequence logo for the TF binding motifs of RUNX3 and STAT5B have been determined from PMMs to overlap rs11811536 while rs12032504 overlaps motifs for TFs NDF2 and Z280D (not shown)

Supplementary Figure S4

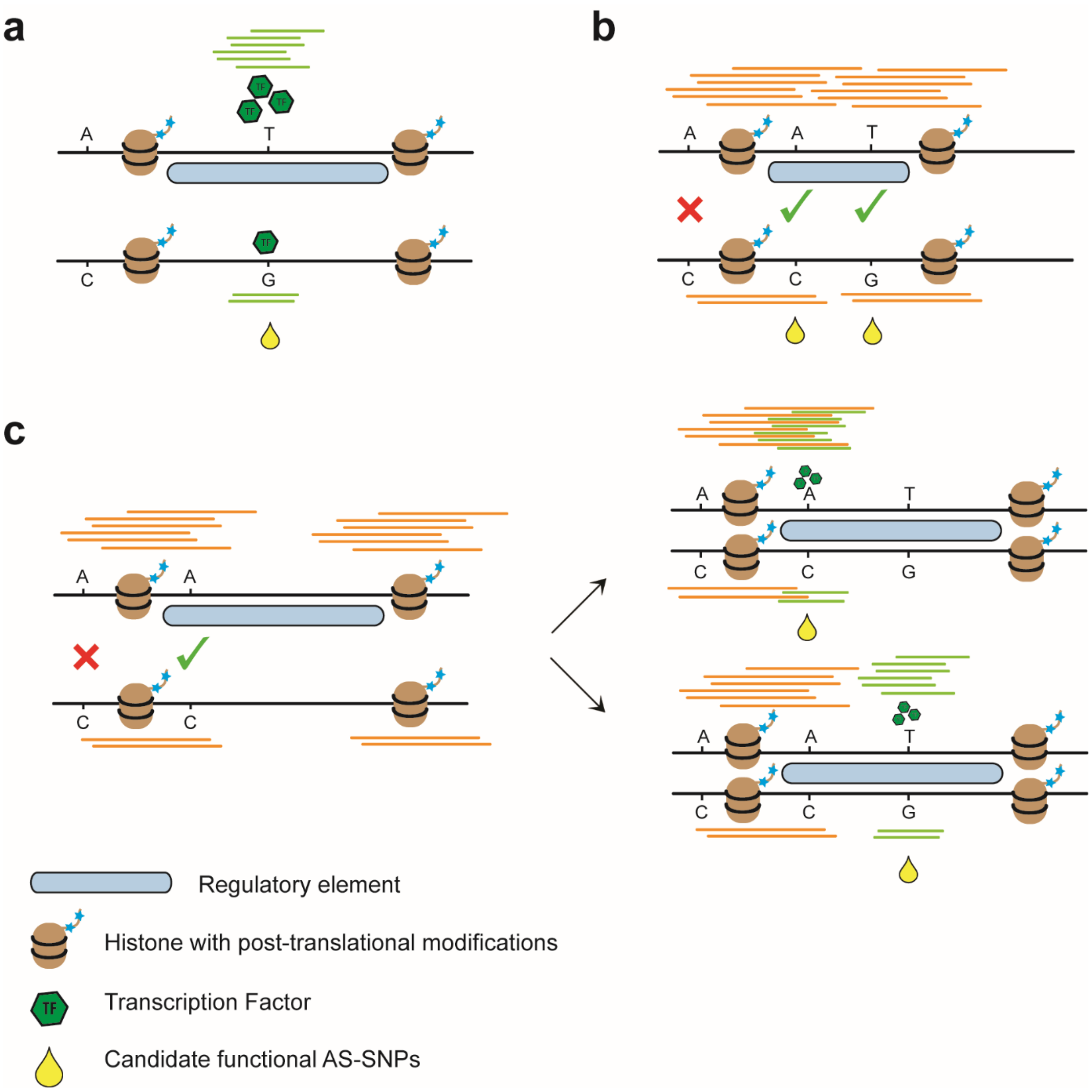

Figure. S4 Different nature of the AS signals obtained using raw reads from ChIP-seq experiments **(a)**. AS-SNP defined by ChIP-seq reads of a TF. **(b)**. AS-SNPs defined by ChIP-seq reads of histone modifications for short regulatory elements (up to ~200 bp). **(c)**. AS-SNPs defined by ChIP-seq reads of histone modifications for longer regulatory elements (> 300 bp). Using shorter reads or investigating longer elements like promoters will not provide a complete coverage of the regulatory element and in this case the defined AS-SNPs would either represent the real functional variant (**c** top) or be the echo of an AS event happening elsewhere in the regulatory element (**c** bottom).
